# Clinical characteristics of distal gastric cancer in young adults from Northeastern Brazil

**DOI:** 10.1186/s12885-018-3995-4

**Published:** 2018-02-05

**Authors:** Manuel B. Braga-Neto, Jessica Gomes Carneiro, Alzira M. de Castro Barbosa, Igor S. Silva, Danielle C. Maia, Felipe S. Maciel, Rafael Jorge Alves de Alcântara, Paulo Roberto L. Vasconscelos, Lucia L. B. C. Braga

**Affiliations:** 1 Department of Internal Medicine, Federal University of Ceará, Fortaleza, CE Brazil; 2 Clinical Research Unit, Federal University of Ceará, Fortaleza, CE Brazil; 3 Department of Surgery, Federal University of Ceará, Fortaleza, CE Brazil; 4 Hospital Universitário Walter Cantídio, Federal University of Ceará, Fortaleza, CE Brazil

**Keywords:** Distal gastric cancer, Young adults, Risk factors, Brazil

## Abstract

**Background:**

It has been suggested that distal gastric carcinoma (GC) in younger patients has a more aggressive outcome than in older patients, however this is a controversial issue. The aim of this study was to compare clinicopathological features between younger and older patients with GC in Northeastern Brazil.

**Methods:**

A total of 207 patients with distal GC (41 patients ≤45 years, considered younger group, and 166 > 45 years, considered older group) were evaluated prospectively during a 6 year period.

**Results:**

The mean patient age in the young group was 37.41 years old and 64.43 years in the older group. No significant difference was found regarding gender, area of residence, history of alcohol consumption, chronic tobacco smoking. Prevalence of first-degree GC history was 12.5% (7.3% in younger group vs. 13.9% in older; *p* <  0.46). The most frequent symptom was gastric pain and weight loss. Diffuse infiltrative cancer was more frequently seen in younger patients (70.70% vs. 33.70%, respectively; *p* <  0.01), as was histologically less differentiated tumors (63.40% vs. 33.10%; *p* <  0.01) and stage IV of GC (48.80% vs. 30.70%; *p* <  0.015). Five-year survival, evaluated in 82 patients, was lower in younger patients (*p* = 0.045); however, after adjusting for stage of GC in the multivariate analysis, this association did not remain significant. Family history of GC and gender had no impact on survival.

**Conclusions:**

Younger patients showed higher prevalence of diffuse type of Lauren and lower survival that was attributed to higher rate of advanced stage of GC. Gastric cancer screening strategies should also be considered in younger individuals, especially in areas of high prevalence. Further studies are warranted to determine risk factors associated with gastric cancer in young adults.

## Background

Gastric cancer (GC) is the third most common cause of cancer-related deaths in the world [1, 2]. Its incidence varies widely among different geographic areas and is thought to cause a higher burden in developing countries than in industrialized nations [2]. Gastric cancer is more common in older patients, with mean age ranging between 50 and 70 years [1, 2]. Although it is considered a rare disease in young individuals, some studies have shown that 2-15% of GC cases are diagnosed in individuals 45 years old or less [3–6].

Several studies have suggested that younger patients may have distinct disease characteristics [4, 5, 7, 8]. Young patients often present with more advanced GC stage, possibly as a result of delayed diagnosis, and have higher rates of histologically undifferentiated tumors, which have been demonstrated in different patient populations. Furthermore, some studies have reported a more aggressive biological pattern with more rapid disease progression and worse prognosis in young GC patients than in middle-age patients [9], although other studies did not find such an association [4–6, 10].

The epidemiology of GC has been widely studied in Japan and in the developed western world [11–13], but only few reports from the developing countries have been published, especially in younger patients [14].

Several factors have been associated with higher risk of gastric cancer such as diets rich in salted, smoked, or poorly preserved foods, tobacco, alcohol, *H. pylori* infection, specially more virulent strains, and positive family history of gastric cancer [15]. The incidence of distal gastric cancer is higher in developing countries, and is probably due to the higher rates of *H. pylori* infection [16].

In Brazil, the incidence of gastric cancer varies from region to region and while it has declined in Midwest, South, Southeast, it has increased in the Northeast of the country [17]. The state of Ceara, located in Northeastern Brazil, has the third highest prevalence of gastric cancer among males in the country and the highest among females [18]. In addition, it has been shown that approximately 10% of patients with GC are 45 years old or less [19]. The prevalence of *H. pylori* infection in state of Ceara is high, at approximately 80% in dyspeptic patients [20] as well as in asymptomatic individuals from the community [21]. The infection is acquired early in childhood [22] and gastric cancer is significantly associated with more virulent strains, such as *cagA* (cytotoxin-associated gene A), of *H. pylori* [23].

Therefore, the purpose of the present study was to evaluate the clinical pathologic characteristics and risk factors associated with distal gastric cancer as well as survival in young patients, defined as 45 years old or less, in comparison with patients above 45 years old, in a referral center of Fortaleza, in the Northeast of Brazil.

## Methods

This study was a prospective epidemiological cohort followed during a 6 year period (2008 to 2014), conducted in a tertiary referral center, Walter Cantidio University Hospital, in Fortaleza, state of Ceara-Brazil. The study was approved by the Institution’s Ethics Committee and all patients signed an Informed Consent Form.

We included in this study patients with gastric adenocarcinoma confirmed by histopathology. Patients with gastroesophageal junction tumors, non-Hodgkin gastric lymphoma or gastrointestinal stromal tumors were excluded. Young GC patients were defined as individuals that were 45 years old or less at time of diagnosis, as reported previously [3–5]. Two groups of patients were set for analyses purposes, young age gastric cancer (≤45 years) and older (> 45 years). A total of two hundred and seven patients were included in this study. Patients were admitted based on Hospital availability through the Public Health System (Sistema Único de Saude - SUS) without any bias related to patient gender or area of residence. This health system provides care to mostly low-income patients and most of them have similar ethnic backgrounds. The patients answered a questionnaire about clinical symptoms, demographic data (age at the time of GC diagnosis, gender and area of residence), alcohol and tobacco use and time of onset of symptoms. A positive family history of GC was defined as a self-reported history of gastric carcinoma among at least one first-degree relative.

The histological criteria of Lauren (intestinal, diffuse, or mixed) was used to classify the gastric adenocarcinoma [24]. The staging of gastric cancer was done in conformity with the tumor, node, metastasis system (TNM), suggested by the American Joint Committee on Cancer [25].

## Statistical analysis

Data were analyzed using the software SPSS (version 16.0, Chicago, IL). Clinicopathologic data were compared using the χ^2^ and Fisher’s exact tests and *p* < 0.05 was considered statistically significant. Patient survival was evaluated with the Kaplan–Meier method and a log-rank test was used to assess differences between groups. The hazard ratio and confidence intervals were estimated using the Cox univariable model and multivariate Cox proportional hazards regression models. Survival was calculated from the date of operation to the date of the most recent follow-up examination or to the date of death. The power of the survival sample was analyzed by GPower Version 3.1.9.2, Germany, 1992-2014).

## Results

### Patient population

Two hundred and seven patients with distal cancer were analyzed, 41 were ≤45 years and 166 were > 45 years old. The mean age for the young group was 37.4 years old (23 – 45 years) and the older group 64.4 years (46 – 86 years).

The demographic and social features of the 207 patients are shown in Table 1.

**Table 1:** Clinicopathological characteristics of patients with distal gastric cancer according to age groups

Variables	≤ 45 years (*n* = 41)		> 45 years (*n* = 166)		*p*
	*n*	(%)	*n*	(%)	
Gender					
Male	25	61.0	113	68.1	0.39
Female	16	39.0	53	31.9	
Residence					
Metropolitan region	30	73.2	104	62.7	0.21
County	11	26.8	62	37.3	
Chronic tobacco history					
Yes	18	43.9	98	59.4	0.08
No	23	56.1	68	40.6	
Alcohol consumption history					
Yes	23	56.1	82	49.4	0.48
No	18	43.9	84	50.6	
Family history of gastric cancer					
Yes	3	7.3	23	13.9	0.46
No	38	92.7	143	85.5	
Gastrectomy					
Curative	24	58.5	107	64.5	< 0.001
Paliative	8	19.5	57	34.3	
No resection	9	22.0	2	1.2	
Lauren type					
Diffuse	29	70.70	56	33.70	< 0.001
Intestinal	9	22.00	101	60.80	
Mixed	3	7.30	9	5.40	
TNM Stage					
I	9	22.00	27	16.30	0.015
II	2	4.90	43	25.90	
III	10	24.40	45	27.10	
IV	20	48.80	51	30.70	
Diferentiation of the tumor					
Well differentiated	0	0.00	6	3.60	< 0.001
Moderately differentiated	13	31.70	105	63.30	
Poorly differentiated	26	63.40	55	33.10	
Undifferentiated	2	4.90	0	0.00	

Overall, 66.0% of patients were males, without statistical difference between age groups (61.0% ≤45 years vs. 68.1 >  45 years). The male to female sex ratio was 1.56/1.00 amongst young patients and 2.13/1.00 in older patients. There was a higher proportion of chronic tobacco smoking among the older subjects (59.4% in patients > 45 years vs. 43.9% in patients ≤45 years, *p* < 0.08), although not statistically significant.

The overall prevalence of alcohol consumption was 50.72% (56.1% in the younger group and 49.4% in older group, *p* = 0.48). Regarding positive family history of GC in the first degree relatives, the overall prevalence was 12.5%, (7.3% of younger patients vs. 13.9% in older group, *p* < 0.46).

The most frequent symptom was abdominal pain followed by weight loss in both groups. Jaundice was present in 12.20% vs. 2.40%, respectively, younger and older group (*p* = 0.006). Ascites was more frequent in younger patients, although no statistical difference was found (Table 2). The average duration of symptoms from onset to diagnosis was 16 months in the cases and 13 months in the controls.

**Table 2:** Symptoms feature of gastric cancer patients, according to age group

	Age ≤ 45y (*n* = 41)		Age > 45 (*n* = 166)		*p*
	*n*	%	*n*	%	
Abdominal pain	37	90.2	135	81.3	0.172
Anemia	22	53.7	111	66.9	0.114
Hematemesis	10	24.4	42	25.3	0.580
Melena	9	21.9	45	27.1	0.500
Weight loss	31	75.6	137	82.5	0.310
Vomit	27	65.9	128	77.1	0.136
Ascites	6	14.6	19	11.5	0.574
Jaundice	5	12.2	4	2.4	0.006

### Histological classification

With regard to Lauren classification, diffusely infiltrative cancer was more frequent in younger than older GC patients (70.70% vs. 33.70%, respectively; *p* < 0.001), even when gender, tobacco smoking, alcohol consumption, family history of gastric cancer were taken into account in multivariate analysis (*p* = 0.001), OR 3.448 CI 95%; 1.681- 7.075. The intestinal type was more frequent in older than in younger GC patients (60.8% > 45 years vs. 22% ≤45 years, respectively; *p* < 0.001).

Poorly differentiated adenocarcinoma was more prevalent in the younger than in older GC patients (63.4% ≤45 years vs. 33.10% > 45 years, *p* < 0.001). On the other hand, moderately differentiated adenocarcinoma was more frequent in older subjects (63.3% of patients > 45 years vs. 31.7% ≤ 45 years; *p* = 0.003), as seen in Table 1.

### Staging

No patients were diagnosed with early GC; almost one-third of the patients had advanced GC. Stage IV of the TNM classification was more frequently observed in the younger group (48.8% ≤45 years vs. 30.7% > 45 years; *p* = 0.015).

Surgical resection was not performed in 22.0% (9/41) of patients ≤45 years versus 1.2% (2/166) of patients > 45 years older due to advanced stage of the tumor (*p* < 0.001).

Regarding the location of the tumor, 37.9% were found in the antrum and 20.8% in the body of stomach. Bormann III was described in 53.66% of younger group patients and 46.39% in older group, respectively (*p* = 0.60).

### Survival characteristics

**Fig. 1:**
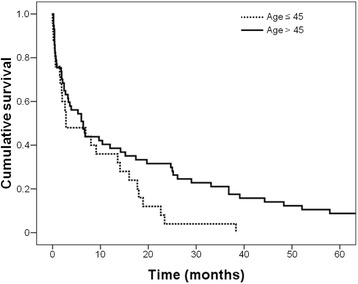
Survival curves of gastric cancer patients according to age groups (*p* = 0.045)

**Fig. 2:**
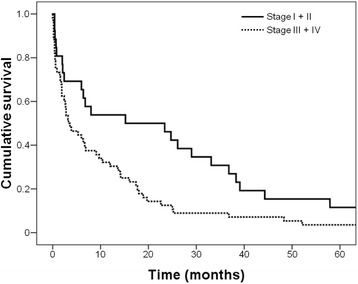
Survival of gastric cancer patients according to stage of disease (*p* = 0.011)

**Fig. 3:**
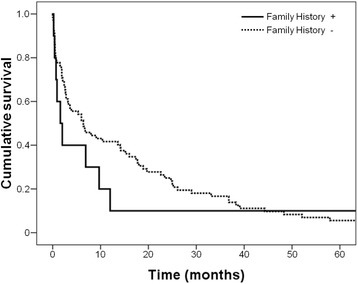
Survival of gastric cancer patients according to family history of gastric cancer (*p* = 0.37)

Survival data was only available in 82 (25 young and 57 older patients) patients due to loss to follow-up. Information up to 60 months after diagnosis was obtained. The group of patients that were lost to follow-up was similar to group with survival data in regards to gender, chronic tobacco, alcohol consumption, TNM stage and histopathologic type of tumor and was different in regards to age and Lauren type. Table 3 shows clinicopathological features of group of GC with survival information. The survival mean for all patients was 16 months (9 months for younger group and 21 months to older group). Fifty six percent (14/25) of young GC patients and 52% (39/57) of old GC patients died within 6 months of follow up. From 6 months to 2 years of follow up, 40% (10/25) vs 17.5% (10/57) died, respectively. One young GC patient survived more than 2 years (4.0%) vs 17 patients in the older GC group (29.8%). Seven patients in the old gastric cancer group (12%) were alive at 60 months. The power of the survival analysis was 0.79 and was calculated taking into account age (the 75 GC patients that died were included in the analysis). There was significant difference in survival between younger and older patients (*p* = 0.045), (Fig. 1). However, after adjusting for stage of GC, gender, family history of GC in the multivariate analysis, this difference did not remain significant (*p* = 0.111), as shown in Table 2. Regarding the stage of GC, a significant difference between survival and stage of GC was found, regardless of age, gender and family history of gastric cancer (hazard ratio: 1.790 CI 1.078 - 2.973; *p* = 0.024), Fig. 2 and Table 4. Positive family history of gastric cancer (mean survival of 14 months for patients with positive family history vs. 17 months for negative family history; *p* = 0.376), (Fig. 3), gender (mean survival 16 months for male vs. 19 months for female; *p* = 0.998) and Lauren type of GC (median survival of 14 months for diffuse vs. 19 months for intestinal vs. 21 months for mixed; *p* = 0.405) did not change survival.

**Table 3:** Characteristics of gastric cancer patients who had survival evaluated according to age groups

Characteristics	Age ≤ 45 years (*n* = 25)		Age > 45ears (*n* = 57)		*p*
	*n*	%	*n*	%	
Gender					
Male	13	52.0	40	70.2	0.113
Female	12	48.0	17	29.8	
Residence					
Metropolitan Region	20	80.0	44	77.2	0.777
County	5	20.0	13	22.8	
Chronic tobacco					
Yes	10	40.0	36	63.2	0.004
No	15	60.0	21	36.8	
Alcohol consumption					
Yes	12	48.0	26	45.6	0.841
No	13	52.0	31	54.4	
History of Gastric Cancer					
Yes	3	12.0	7	12.3	0.971
No	22	88.0	50	87.7	
Gastrectomy					
Curative	12	80.0	24	63.2	0.237
Paliative	3	20.0	14	36.8	
Lauren type					
Diffuse + Mixed	21	84.0	27	47.4	0.001
Intestinal	4	16.0	30	52.6	
TNM					
I + II	4	16.0	22	38.6	0.043
III + IV	21	84.0	35	61.4	
Histological type					
Well differentiated	0	0.0	2	3.5	0.003
Moderately differentiated	5	20.0	33	57.9	
Poorly differentiated	19	76.0	22	38.6	
Undifferentiated	1	4.0	0	0.0

**Table 4:** Survival analyzes of 82 patients after primary gastric cancer resection

Variables	Univariable		Multivariable	
	Harzard ratio	p*	Harzard ratio	p*
Age (> 45 vs. ≤ 45 years)	1.647 (1.002, 2.707)	0.049	1.507 (0.910, 2.496)	0.111
Gender (female vs. male)	1.000 (0.622, 1.608)	0.998		
Histology (diffuse vs. intestinal)	0.924 (0.586, 1.458)	0.735		
Family History (positive vs. negative)	0.729 (0.362, 1.468)	0.376		
Stage (III-IV vs. I-II)	1.885 (1.143, 3.108)	0.013	1.790 (1.078, 2.973)	0.024

Values in parentheses are 95% confidence intervals. *Cox proportional hazards analysis

## Discussion

Gastric cancer is a relevant public health problem, although the incidence and mortality rates have decreased in the last 30 years [26]. This trend has also been seen in Brazil overall, except in Northeast of the country where it has actually been increasing [17]. In some regions such as USA and China, although the overall incidence of GC has been declined, studies have suggested that it is increasing in the younger patient population [7, 27].

It remains unclear whether GC in young patients differs from the older patients in terms of biological and clinical behavior. The prevalence of gastric cancer is about twice as high among men than women [28]; nevertheless, patients under 45 years had been reported to have a higher prevalence of GC in women [5, 28]. In the present study, the prevalence of gastric cancer was higher in males without statistical difference between age groups, however male to female sex ratio was 1.56/1,00 among young patients and 2.13/1,00 in older patients, in agreement with a study from Turkey [29].

Tobacco smoking is a risk factor for gastric adenocarcinoma, especially when tumor is located in the cardia [30]. There appears to be a causal relationship between smoking and gastric cancer, as demonstrated in a large cohort involving several European countries [31]. In this study, positive smoking history was present in approximately half of the patients and was more prevalent in older gastric cancer patients, however, without statistical difference.

Several studies have shown that stomach cancer tends to aggregate among family members [32, 33]. In the present study, the overall prevalence of positive history of gastric cancer in first-degree relatives was 12.56% (7.3% in young group and 13.90% in old group, without significant statistical difference). This results are higher than what has been reported in young vs. older patients in studies from Italy (5% vs. 6.2%, respectively) [28], Japan (5.9% vs. 6.3%) [6], however lower than the prevalence reported from China (25% vs. 16%) [7] and Mexico (15.40% vs. 2.60%) [14].

The familial clustering of gastric cancer may be explained by the combination of factors since relatives of gastric cancer patients share not only similar genetic background, which controls the inflammatory responses, but also environmental and lifestyle factors. It has been postulated that one major environmental factor could be *H. pylori* infection [32], since several studies have demonstrated that *H. pylori* infection cluster within families, and it may often be transmitted from parents to their children in early childhood as well as between siblings [34]. Previously we have reported that the prevalence of *H. pylori* infections among the first degree relatives of gastric cancer is similar to dyspeptic patient from the same economic level; however, the relatives of gastric cancer had higher incidence of precancerous lesions [20] and were colonized with more virulent strains [35]. In addition, most GC patients were infected with *H. pylori* and *cagA* strains were significantly associated with GC [23].

It has been shown that the diffuse type of gastric cancer was more prevalent in younger than in older patients [5, 7, 36–38]. In this study, age under 45 years old was significantly associated with diffuse gastric cancer even when adjusting for gender, tobacco smoking, alcohol consumption and family history of gastric cancer in multivariate analysis. It is not well understood why the majority of young patients have diffuse type of GC, which is a more aggressive tumor. Molecular differences between gastric carcinomas of young and older patients have been studied, with discrepant results. A Mexican study found that polymorphisms of E-cadherin gene was associated with diffuse gastric cancer in young patients [37]. Furthermore, a study from Korea showed that diffuse GC in young patients had higher proportion of CDH1 alterations and was associated with shorter survival, suggesting that this may contribute to more aggressive clinical course of in young GC patients [8].Conversely, a study from Iran, did not find any differences in the expression of E-cadherin and Syndecan-1, cell adhesion molecules [38].

Several studies have reported that the majority of gastric cancer patients were diagnosed at stage III or IV [6, 10]. Overall, the survival rates of GC in this study were low, similar to what was found in a study conducted in Southeastern Brazil [39]. In the present study almost one third of the patients had advanced GC (TNM stages III + I) and none of the patients were diagnosed with early GC, defined as invasion confined to either the mucosa or submucosa, irrespective of lymph node metastasis, according to the Japanese Society of Gastroenterological Endoscopy [40]. The younger group was associated with advanced stage of the disease and nearly half of them were stage IV. In addition, a higher number of patients in this group were considered inoperable due to spreading of the GC. This finding is in agreement with studies from Japan in which a worse prognosis due to high prevalence of stage IV and peritoneal dissemination was found in young patients [41]. In contrast, a study from Mexico did not find significant differences in clinicopathological feature of GC between young and elderly patients [14].

It remains unclear why younger patients present in a more advanced stage. Perhaps lack of awareness regarding disease and delay in seeking medical attention could be contributing factors. Colonization with more virulent strains of *H. pylori* may also be another important factor that should be evaluated in future studies.

The symptoms of GC are nonspecific and vague in the earlier stage of disease, and most of the time when patients report weight loss or obstructive symptoms, they are often already in an advanced stage, precluding curative radical resection. This fact might contribute to the delay in GC diagnosis as well as the poor prognosis that is observed in GC patients overall. In the present study, the most prevalent symptom was abdominal pain, followed by weight loss (without difference between age groups), while jaundice was significantly more present in the younger group. These findings are in agreement with others reports [29]. Only jaundice was significantly more common in the younger group.

In this study, the 5 year survival of young patients with GC was significantly lower than in older patients, however after adjusting for stage of GC in the multivariate analysis, this association was not significant. Furthermore, advanced stage of the disease was associated with worse survival regardless of age, in agreement with other studies [6, 10]. It has been reported that young GC patients who undergo curative resection do not have a worse prognosis than older patients [5, 6, 10], with some studies reporting that survival rates in this group was actually better [4, 10]. Family history of GC, gender, and Lauren type of GC did not impact survival, which is an contrast with a study by Medina Franco et al., that reported a significant association of family history of GC with poor survival [14].

Strengths of this study include its prospective design as well as similar population background regarding social economic level, ethnicity and access to care. This study has several limitations such as small sample size, follow-up period limited to 5 years and survival information only available in a small number of patients.

## Conclusions

In summary, this study found a significant age-specific difference in the clinical and pathological features among patients with GC. Younger patients had a high frequency of diffuse type of Lauren and advanced stage of GC. Overall, younger patients had lower survival rates when compared to patients above 45 years old, however this association was not significant after adjusting for the stage of GC. Strategies to improve early detection of gastric cancer should also include younger patient population, especially in geographic areas where prevalence is high. Further studies are warranted in order to better understand age difference in gastric cancer behavior as well as potential associated risk factors with gastric cancer in younger individuals, including *H. pylori* status and strain virulence.

## Abbreviations

*cagA*: cytotoxin-associated gene A
GC: Gastric cancer
*H. pylori*: *Helicobacter pylori*
vs: versus
